# Structure-based site-directed photo-crosslinking analyses of multimeric cell-adhesive interactions of voltage-gated sodium channel β subunits

**DOI:** 10.1038/srep26618

**Published:** 2016-05-24

**Authors:** Hideaki Shimizu, Haruko Miyazaki, Noboru Ohsawa, Shisako Shoji, Yoshiko Ishizuka-Katsura, Asako Tosaki, Fumitaka Oyama, Takaho Terada, Kensaku Sakamoto, Mikako Shirouzu, Shun-ichi Sekine, Nobuyuki Nukina, Shigeyuki Yokoyama

**Affiliations:** 1 RIKEN Systems and Structural Biology Center, Tsurumi, Yokohama 230-0045, Japan; 2 RIKEN Center for Life Science Technologies, Tsurumi, Yokohama 230-0045, Japan; 3 Laboratory for Structural Neuropathology, RIKEN Brain Science Institute, Wako, Saitama 351-0198, Japan; 4 Department of Neuroscience for Neurodegenerative Disorders, Juntendo University Graduate School of Medicine, Tokyo 113-8421, Japan; 5 Laboratory of Structural Neuropathology, Doshisha University Graduate School of Brain Science, 1-3 Tatara Miyakodani, Kyotanabe-shi, Kyoto 610-0394, Japan; 6 Department of Chemistry and Life Science, Kogakuin University, Hachioji, Tokyo 192-0015, Japan; 7 RIKEN Structural Biology Laboratory, Tsurumi, Yokohama 230-0045, Japan

## Abstract

The β1, β2, and β4 subunits of voltage-gated sodium channels reportedly function as cell adhesion molecules. The present crystallographic analysis of the β4 extracellular domain revealed an antiparallel arrangement of the β4 molecules in the crystal lattice. The interface between the two antiparallel β4 molecules is asymmetric, and results in a multimeric assembly. Structure-based mutagenesis and site-directed photo-crosslinking analyses of the β4-mediated cell-cell adhesion revealed that the interface between the antiparallel β4 molecules corresponds to that in the *trans* homophilic interaction for the multimeric assembly of β4 in cell-cell adhesion. This *trans* interaction mode is also employed in the β1-mediated cell-cell adhesion. Moreover, the β1 gene mutations associated with generalized epilepsy with febrile seizures plus (GEFS+) impaired the β1-mediated cell-cell adhesion, which should underlie the GEFS+ pathogenesis. Thus, the structural basis for the β-subunit-mediated cell-cell adhesion has been established.

Voltage-gated sodium channels are transmembrane proteins responsible for the generation of action potentials, and play important roles in the modulation of electrical impulses^1^. They consist of a pore-forming α subunit and one or more auxiliary β subunits. To date, five β subunits (β1/β1B–β4, encoded by *SCN1B–4B*, respectively) have been identified. Each β subunit has an extracellular immunoglobulin (Ig) domain, a transmembrane domain, and an intracellular domain, except for the secreted form of β1B^2^.

Furthermore, the β subunits function as cell adhesion molecules (CAMs)^3^. The β1, β2, and β4 subunits mediate their *trans* homophilic and heterophilic interactions^2^. In the peripheral and central nervous systems, the β subunits are expressed on both axons and glial cells^4^,^5^, and in *SCN1B* (−/−) mice, the contacts between axons and glial cells are disrupted in a subset of axons^6^. Therefore, the β subunits serve as CAMs through the interactions of their β subunit extracellular domains with themselves, other CAMs, and extracellular matrix proteins, probably around the junctions of the nodes of Ranvier and axon initial segments with astrocytes and Schwann cells^2^. Furthermore, the CAM activities of the β subunits are important for the regulation of neuronal migration, pathfinding, and fasciculation^7^. We recently found the diffuse distribution of β4 along the unmyelinated axons in striatal projection fibers, suggesting that β4 might participate in cell-cell adhesion in these fibers^8^. However, the interfaces for the trans interactions of the β subunits have remained elusive, in spite of their importance in cell-cell adhesion.

The β subunits play a crucial role in the pathogenesis of epilepsy, which is associated with the abnormal modulation of voltage-gated sodium channels^7^. The generalized epilepsy with febrile seizures plus (GEFS+) syndrome has been correlated directly with mutations in *SCN1B*. Four mutations (C121W, R85H, R85C, and I70_E74del) have been identified, and all of them are located in the extracellular domain of β1^9^,^10^,^11^. In heterologous expression studies, the C121W mutant was expressed on the cell surface and promoted the cell surface expression of the α subunit, but exhibited impaired β1–β1 homophilic cell adhesion^12^,^13^. However, it is unclear why the C121W mutant lacks the adhesive activity, and whether the other GEFS+ mutations besides C121W impair the β1–β1 homophilic cell adhesion.

In the present study, we determined the structure of the β4 subunit extracellular domain fragment by X-ray crystallography, and identified an interface for antiparallel homophilic interactions in the crystal lattice. By structure-based mutagenesis and photo-crosslinking experiments combined with cell aggregation assays, we revealed that the identified interface is actually important for the *trans* homophilic interaction in the β4-mediated cell-cell adhesion, in the native environment of living cells. Furthermore, the corresponding interface of β1 includes or adjoins the GEFS+ mutations, and all of these mutations were confirmed to disrupt the β1-mediated *trans* homophilic interaction in cell-cell adhesion. Thus, our findings establish the structural basis for the *trans* homophilic interaction of the β subunits, in relation to their cellular functions and GEFS+ pathogenesis.

## Results

### The antiparallel arrangement of β4 in the crystal lattice

**Figure 1:**
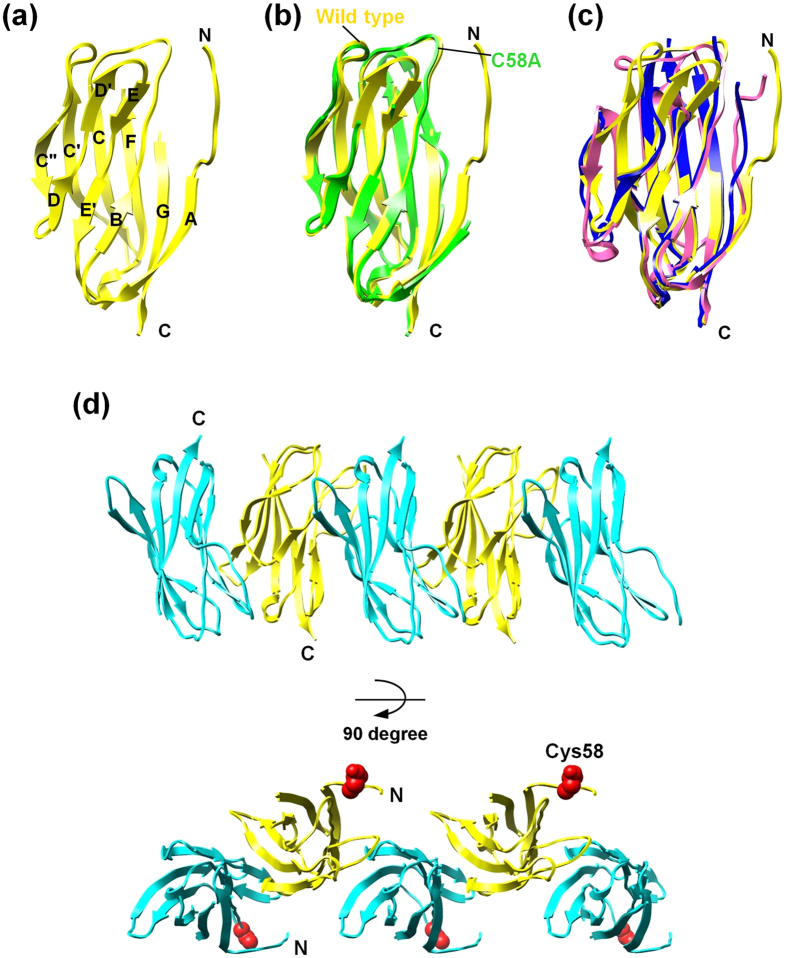
Crystal structure of the β4 subunit extracellular domain. (**a**) Ribbon representation of the β4 structure. The β strands are represented as arrows, and labeled according to the standard convention used for the immunoglobulin fold^36^. (**b**) Comparison of the structures of β4ex (yellow) and the C58A mutant (4MZ2, green). (**c**) Comparison of the structures of β4ex (yellow), β2ex (5FEB, purple), and β3ex (4L1D, blue). (**d**) Molecular arrangement in the crystal packing. The β4 molecules are colored yellow and blue, and are in the same orientation. Side view (upper) and top view (lower).

We determined the structure of the mouse β4 subunit extracellular domain fragment (β4ex) by X-ray crystallography. Unlike the human β4 subunit extracellular domain fragment used in the previous crystallographic study, which had either the C58A or C131W mutation^14^, the present fragment contains all three of the Cys residues. The molecular structure of β4ex is highly superimposable on that of the C58A mutant^14^, except for the N-terminal region and the mutated residues (Fig. 1a,b). The N-terminal region of β4 does not participate in the β-sheet of the Ig fold, and appears to be flexible. The N-terminal flexibility appears to be conserved in β2 and β3 (Fig. 1c)^15^,^16^.

**Figure 2:**
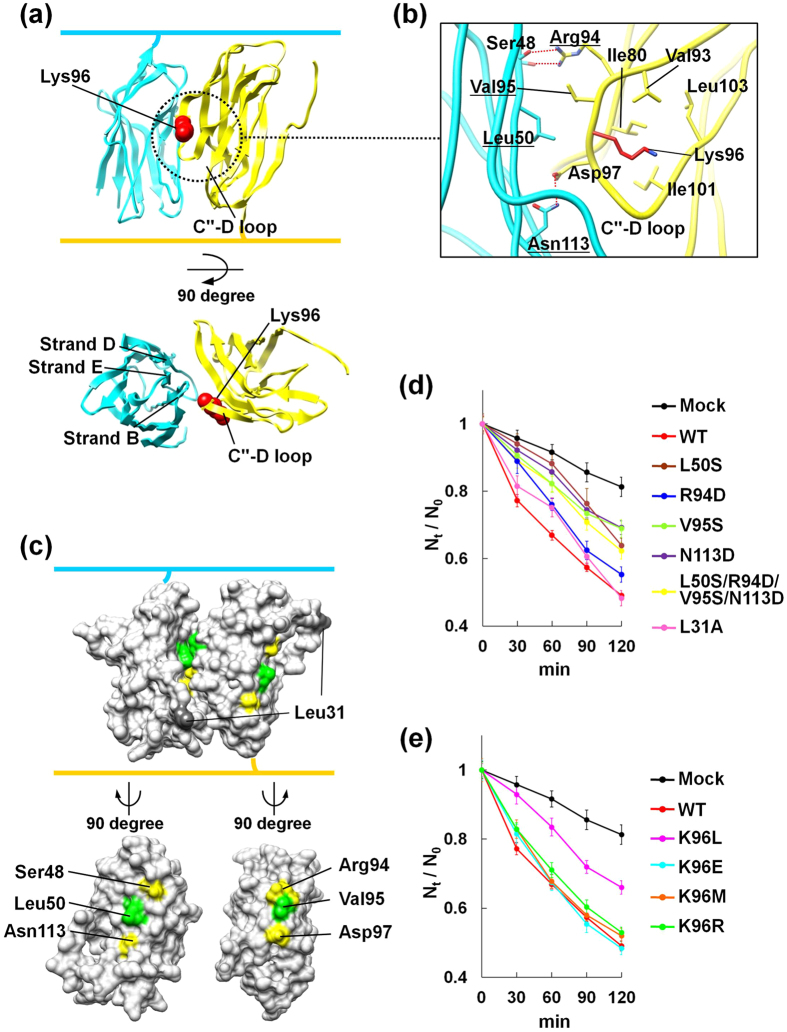
Cell aggregation assay of the CHO cells stably expressing the WT and mutants of β4. (**a**) Ribbon representation of the interactions in the antiparallel arrangement of β4. Side view (upper) and top view (lower). (**b**) Close-up view of the C″–D loop interactions. The side chains of Lys96 are colored red, and the mutated residues in the L50S/R94D/V95S/N113D mutant are underlined. (**c**) Surface representations of the contact areas within the *trans* homophilic interface. The surface-exposed hydrophobic residues are colored green, and hydrophilic residues are yellow. Side view in the same orientation as in Fig. 2a (upper), and view of the contact surface of the *trans* homophilic interface (lower). (**d**) Cell aggregation kinetics. Mock (black), WT (red), L50S (brown), R94D (blue), V95S (light green), N113D (purple), L50S/R94D/V95S/N113D (yellow), and L31A (pink). The aggregation is represented by the index *N*_*t*_/*N*_0_, where *N*_*t*_ and *N*_0_ are the total number of particles at incubation times *t* and 0, respectively. Data are means ± S.E.M., n = 24, from three independent experiments. (**e**) Cell aggregation kinetics. K96L (magenta), K96E (light blue), K96M (orange), and K96R (green). Data are means ± S.E.M., n = 24, from three independent experiments.

The β4ex structure exhibits a remarkable feature: a highly ordered multimeric assembly of β4ex molecules is formed in the crystal lattice, and the β4ex molecules interact with each other in an antiparallel manner (Fig. 1d). The antiparallel arrangement is mediated by the interaction of the C″–D loop of one β4 molecule with strands B, D, and E of the β-sheet composed of strands B, E–E′, and D–D′ of the other β4 molecule (Fig. 2a,b). The side chain of Arg94 in the C″–D loop hydrogen bonds with the side and main chains of Ser48 in strand B. The side chain of Asp97 in the C″–D loop hydrogen bonds with the side chain of Asn113 in strand E. Val95, in the C″–D loop, participates in a hydrophobic interaction with Leu50 in strand B (Fig. 2b,c). This antiparallel interface is asymmetric between the two β4ex molecules, and can successively array the β4ex molecules in a linear manner (Fig. 1d).

### Cell aggregation assay for the *trans* homophilic interaction of β4 in CHO cells

Next, we intended to test the possibility that the observed antiparallel interface between two β4 molecules is relevant to the *trans* homophilic interaction in cell-cell adhesion. A cell aggregation assay was previously performed for β1^12^,^17^. Therefore, we established a CHO cell line stably expressing the full-length wild-type (WT) β4 protein, and performed a cell aggregation assay. The WT-expressing CHO cells showed increased cell aggregation, in comparison with the control cells transfected with the empty vector (mock cells) (Fig. 2d and Supplementary Fig. S2a). A cell surface biotinylation assay revealed that the WT β4 protein was expressed on the extracellular surface of the CHO cells (Supplementary Fig. S2b). These observations suggested that β4 mediates cell-cell adhesion on the surface of CHO cells.

### Effects of mutations within the *trans* homophilic interface of β4 on cell aggregation

We next introduced four different point mutations (L50S, R94D, V95S, and N113D), the quadruple mutation (L50S/R94D/V95S/N113D), and the control mutation (L31A) into the full-length β4 molecule (Fig. 2b,c), to disrupt the putative *trans* homophilic interface. The CHO cells stably expressing the L50S, V95S, N113D, and L50S/R94D/V95S/N113D mutants exhibited less cell aggregation than those expressing the WT, R94D, and control L31A mutants (Fig. 2d). A cell surface biotinylation assay confirmed that all of the mutants were expressed on the surface of CHO cells at the same or higher levels than the WT (Supplementary Fig. S2b). Thus, the decreased cell aggregation observed with the L50S, V95S, N113D, and L50S/R94D/V95S/N113D mutants is due to the disruption of the *trans* homophilic interaction. Comparing the *trans* homophilic interface between mouse and human β4s, the interactions of Leu50-Val95 (Leu95 in human) and Asp97-Asn113 are likely to be conserved, but the interaction of Ser48-Arg94 is not (Glu48-Thr94 in human). It is presumable that the residues at positions 48 and 94 are not involved in the *trans* homophilic interaction, and the R94D mutation therefore has little effect on cell aggregation, as compared to the other mutations (Fig. 2d). Consequently, we concluded that the C″-D loop is involved in the interface for the *trans* homophilic interaction in cell-cell adhesion.

Lys96, which is located in the C″–D loop of β4, corresponds to Arg85 in β1, which is related to the GEFS+ mutation (Fig. 2b and Supplementary Fig. S1). We mutated Lys96 to Leu, Glu, Met, and Arg, and examined the aggregation propensities of CHO cells stably expressing these mutants. Remarkably, the K96L-expressing cells showed less cell aggregation activity than the WT-expressing cells. In contrast, the aggregation degrees of the K96E-, K96M-, and K96R-expressing cells were similar to that of the WT-expressing cells (Fig. 2e). Cell surface biotinylation assays confirmed that the Lys96 mutants were expressed on the cell surface at higher levels than the WT (Supplementary Fig. S2b). These observations clearly indicated that the K96L mutant is less effective for cell-cell adhesion than the WT. As shown in Fig. 2b, the side chain of Lys96 is oriented toward the inside of the C″–D loop, and is surrounded by the hydrophobic residues Ile80, Val93, Ile101, and Leu103. Thus, the introduction of a strong hydrophobic residue, such as a Leu residue, at this position seems to cause a conformational change in the C″–D loop, resulting in a substantial decrease in the *trans* homophilic interaction. Taken together, these results suggested the involvement of the C″–D loop in the *trans* homophilic interaction for cell-cell adhesion, and furthermore highlighted the significance of Lys96.

### Photo-crosslinking analysis of the *trans* homophilic interface of β4 in CHO cells

**Figure 3:**
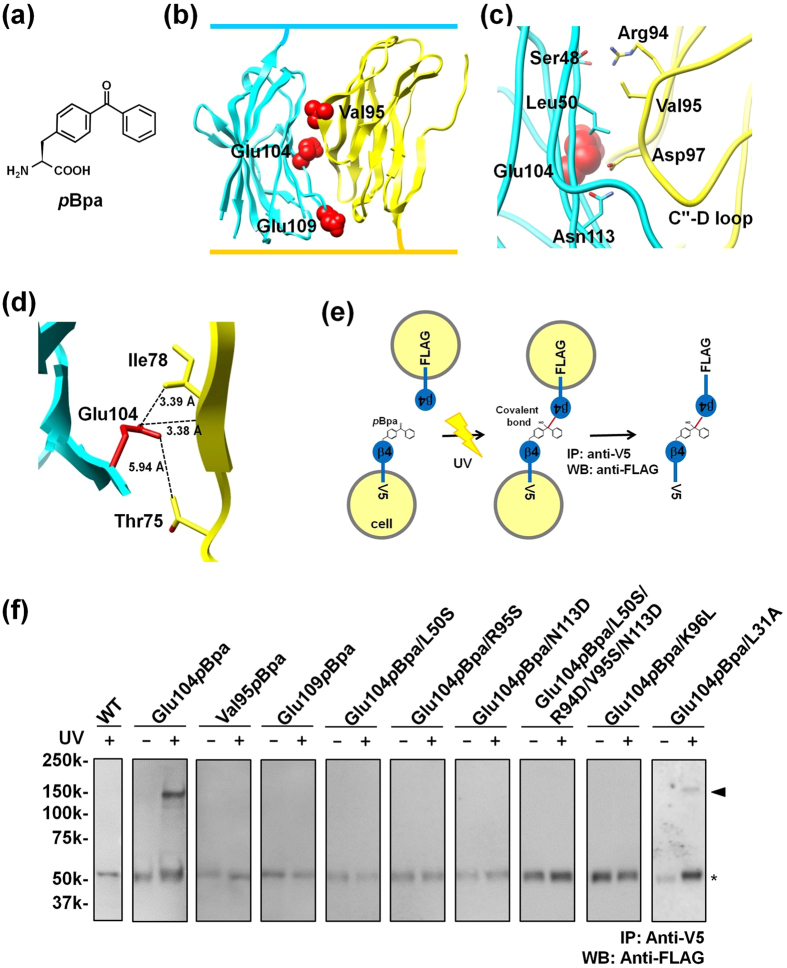
Photo-crosslinking analysis of the *trans* homophilic interaction of β4. (**a**) Chemical structure of *p*Bpa. (**b**) Mapping of Glu104, Val95 and Glu109 substituted by *p*Bpa on the β4 structure. Glu104, Val95 and Glu109 are colored red. (**c**) Close-up view of the C″–D loop interactions and Glu104. (**d**) Close-up view of Glu104, showing the observed distances between the side chain of Glu104 and the carbon atoms in the neighboring β4 molecule. (**e**) Strategy of the photo-crosslinking experiment. The cells separately expressing the *p*Bpa mutants of FLAG- or V5-His-tagged β4 were mixed at a 1:1 ratio, followed by photo-crosslinking. (**f**) Photo-crosslinking of β4. The cell lysates were immunoprecipitated with the anti-V5 antibody, and analyzed by western blotting using an anti-Flag antibody. The triangle indicates the crosslinked β4, and the asterisk indicates the bands corresponding to the anti-V5 antibody, which cross-react with the anti-Flag antibody.

We next planned to examine whether the *trans* interaction of β4 in the present crystal lattice corresponds to that in the homophilic association of β4 for cell adhesion, to obtain another viewpoint in addition to the structure-based mutagenesis. For this purpose, a site-directed photo-crosslinking technique can be used to analyze protein-protein interactions in mammalian cells^18^, and it allows the protein-protein interactions at the interface of interest to be specifically analyzed. In this technique, a photo-reactive non-natural amino acid is inserted in response to an in-frame UAG codon corresponding to the desired site within the protein, with a transfer RNA (tRNA) specific to the UAG stop codon and an engineered aminoacyl-tRNA synthetase specific to the photo-reactive amino acid in the cells^18^,^19^. The photo-reactive amino acid, *p*-benzoyl-L-phenylalanine (*p*Bpa) (Fig. 3a), can be incorporated at or near the interface of the proteins on the basis of the crystal structure, and thus enables the validation of the protein-protein interaction at the interface of interest in the natural cellular environment. In the case of membrane proteins, the site-directed photo-crosslinking analysis of the full-length membrane protein is highly complementary to the crystallographic structural analysis of the extracellular region of the membrane protein. This combination of techniques has successfully been used for analyses of the *cis* interactions of membrane proteins in a cell, *e.g.* a homophilic multimer of CD38^20^ and the CRLR•RAMP2 complex^21^, and confirmed that the intermolecular interactions observed in the crystal packing represent a physiologically relevant form. However, this structure-based photo-crosslinking method has not yet been applied to the *trans* interactions between cells. In this study, we tested this method, for the first time, for the analysis of the *trans* homophilic interaction.

The non-natural amino acid *p*Bpa was used for the site-directed photo-crosslinking. We selected Glu104 as the site of the *p*Bpa substitution (the Glu104*p*Bpa mutant), for the following reasons. This residue is located at the center of the *trans* homophilic interface, but is not directly involved in the *trans* homophilic interaction of β4 (Fig. 3b,c). Thus, it was expected that the incorporation of *p*Bpa at this position would not disrupt the *trans* homophilic interaction of β4. In addition, the Glu104 side chain is located at distances of 3.38–5.94 Å from the carbon atoms in the neighboring β4 molecule (Fig. 3d), and a distance of about 3.3 Å exists between the imaginary phenylalanine and adjacent residues (Supplementary Fig. S3a). Thus, the benzophenone group of *p*Bpa is likely to react with the carbon atoms in its close proximity^18^, and Glu104 is an appropriate site for the *p*Bpa incorporation to detect efficient photo-crosslinking in the *trans* homophilic interaction of β4 between cells. Additionally, the Val95 and Glu109 residues were selected as sites for the *p*Bpa substitution, as putative negative controls. The Val95 residue is involved in the hydrophobic interaction on the *trans* homophilic interface (Fig. 3b,c). The side chain of this residue is very close to the carbon atoms on the interface of the neighboring β4 molecule, with a distance of 0.9–2.0 Å between the imaginary phenylalanine and adjacent residues (Supplementary Fig. S3a). Thus, the substitution of the bulky *p*Bpa for this residue is expected to sterically inhibit the crosslinking or disrupt the *trans* homophilic interaction of β4. The Glu109 residue is located on the edge of the *trans* homophilic interface (Fig. 3b). The side chain of this residue is very close to Glu122 of the neighboring β4 molecule, and is likely to be oriented towards the solvent, with a distance of about 9.0 Å between the imaginary phenylalanine and the adjacent Glu112 residue (Supplementary Fig. S3a). Thus, this residue is not expected to be able to initiate crosslinking.

The genes encoding the full-length β4 Glu104*p*Bpa, Val95*p*Bpa and Glu109*p*Bpa mutants, in which the original codons were replaced by the UAG codon, also encoded either a C-terminal Flag or V5-His tag (Fig. 3e). Two plasmids with differently tagged constructs were individually introduced into CHO cells, together with the genes encoding the tRNA and aminoacyl-tRNA synthetase mutants specific to UAG and *p*Bpa, respectively^18^,^19^. As shown in Supplementary Fig. S3b, the Glu104*p*Bpa, Val95*p*Bpa, and Glu109*p*Bpa mutant proteins were expressed in the presence of *p*Bpa, indicating that *p*Bpa was incorporated at the UAG mutation site of β4. Next, the two CHO transfectants were mixed at a 1:1 ratio, and then subjected to photo-crosslinking and immunoprecipitation with anti-V5, followed by western blotting with anti-Flag (Fig. 3e). In the fraction of the Glu104*p*Bpa mutant immunoprecipitated with anti-V5, the crosslinked product of the β4 multimer was only detected by western blotting, but not under the UV(−) conditions (Fig. 3f). Considering the ability of *p*Bpa to crosslink with its close neighbors, the crosslinking occurs at the center of the *trans* homophilic interface between the adhered β4 proteins. Accordingly, these observations clearly indicated that the *trans* homophilic interface seen in the present crystal lattice is actually involved in the *trans* homophilic interaction of β4 in cell-cell adhesion. The crosslinked product was detected at approximately 150 kDa, indicating that β4 formed multimers. It is conceivable that the adhered β4 proteins may form a multimeric structure similar to that observed in the crystal structure.

### Effects of the *trans* homophilic interface mutations on the β4 photo-crosslinking

Additionally, we introduced the L50S, V95S, N113D, L50S/R94D/V95S/N113D, K96L and L31A mutations used in the cell aggregation assay into the full-length β4 Glu104*p*Bpa mutant, and performed the photo-crosslinking experiment. These proteins were expressed in the presence of *p*Bpa (Supplementary Fig. S3b), and the crosslinked product was not detected in any of the mutants except for the Glu104*p*Bpa/L31A control mutant (Fig. 3f). These observations suggested that all of the mutants, except for the control mutant, disrupt the *trans* homophilic interaction in cell-cell adhesion, in good agreement with the cell aggregation assay results (Fig. 2d), and reasonably strengthened our conclusion that the *trans* homophilic interface identified in this study is involved in cell-cell adhesion.

### The GEFS+ mutations of β1 impair cell-cell adhesion

**Figure 4:**
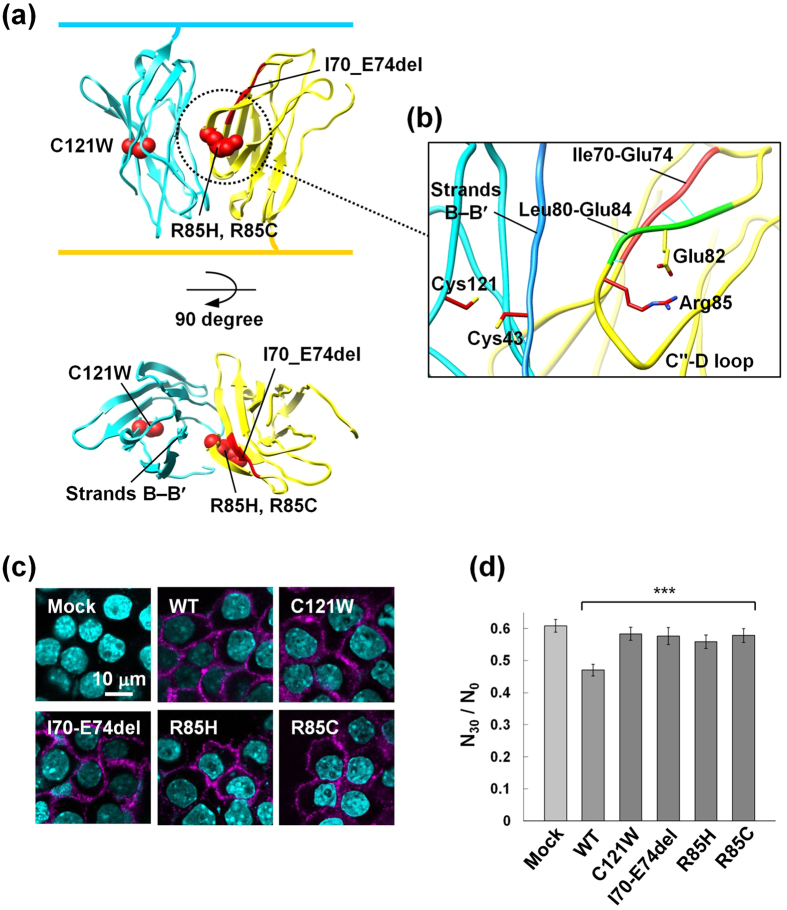
Effects of the GEFS+ mutations on the cell-cell adhesion. (**a**) Mapping of the GEFS+ mutations on the β1 model structure. Side view (upper) and top view (lower). The model was generated by the superimposition of the monomer model (built by the program MODELLER^37^) onto the β4 structure in Fig. 2a. (**b**) Close-up view of the C″–D loop interaction. The hydrogen bonds are indicated by light blue lines. (**c**) Immunofluorescence staining of Neuro2A cells stably expressing the WT and GEFS+ mutants of β1. The β1 proteins were immunostained with anti-β1ex against the N-terminal residues 70–98 of the β1 extracellular domain (Abcam ab107370), and visualized by Alexa 647 (magenta). Nuclei were stained with Hoechst 33342 (cyan). (**d**) Aggregation of Neuro2A cells stably expressing the WT and GEFS+ mutants of β1. Results are expressed as *N*_30_/*N*_0_, where *N*_30_ is the number of particles after a 30 min incubation, and *N*_0_ is the number of particles at the start. ***P < 0.001 (one-way ANOVA, means ± S.E.M, n = 48 from three independent experiments).

All four of the GEFS+ -causing mutations identified in *SCN1B* (C121W, R85H, R85C, and I70_E74del) are located in the extracellular domain of β1 (Fig. 4a,b). The mutation of Lys96 in β4, corresponding to Arg85 in β1 related to the GEFS+ mutation, could disrupt the β4-dependent cell-cell adhesion (Fig. 2e). Moreover, an analysis of the contact areas within the *trans* homophilic interface for the β1 model structure revealed that the pairs of hydrophobic and hydrophilic interactions appear to be present in a similar manner to those in β4, and are likely to mediate the *trans* homophilic interaction of β1 in a comparable mode to that of β4 (Supplementary Fig. S4a). Therefore, we investigated whether the GEFS+ mutations of β1 affected either cell-cell adhesion or cell surface expression. We were unable to establish CHO cell lines stably expressing the full-length β1 protein, for unidentified reasons. Instead, we established Neuro2A cell lines stably expressing the full-length β1 protein. Immunocytochemical experiments of non-permeabilized cells, using anti-β1ex against the N-terminal residues 70–98 of the β1 extracellular domain (Abcam ab107370), and cell surface biotinylation assays indicated that all of the GEFS+ mutants of β1 were expressed on the cell surface of Neuro2A cells (Fig. 4c and Supplementary Fig. S4b,c). In addition, the Neuro2A cells stably expressing the GEFS+ mutants showed decreased cell aggregation, as compared to those expressing WT β1 (Fig. 4d and Supplementary Fig. S4d). Presumably, therefore, the *trans* homophilic interaction mode (Fig. 4a), modeled according to that of β4, is also involved in cell-cell adhesion mediated by β1, and is impaired by the GEFS+ mutations. This is in agreement with the previous findings that the C121W mutation was expressed on the cell surface, and disrupted the cell-cell adhesion^12^,^13^.

### Photo-crosslinking analysis of the *trans* homophilic interface of β1

**Figure 5:**
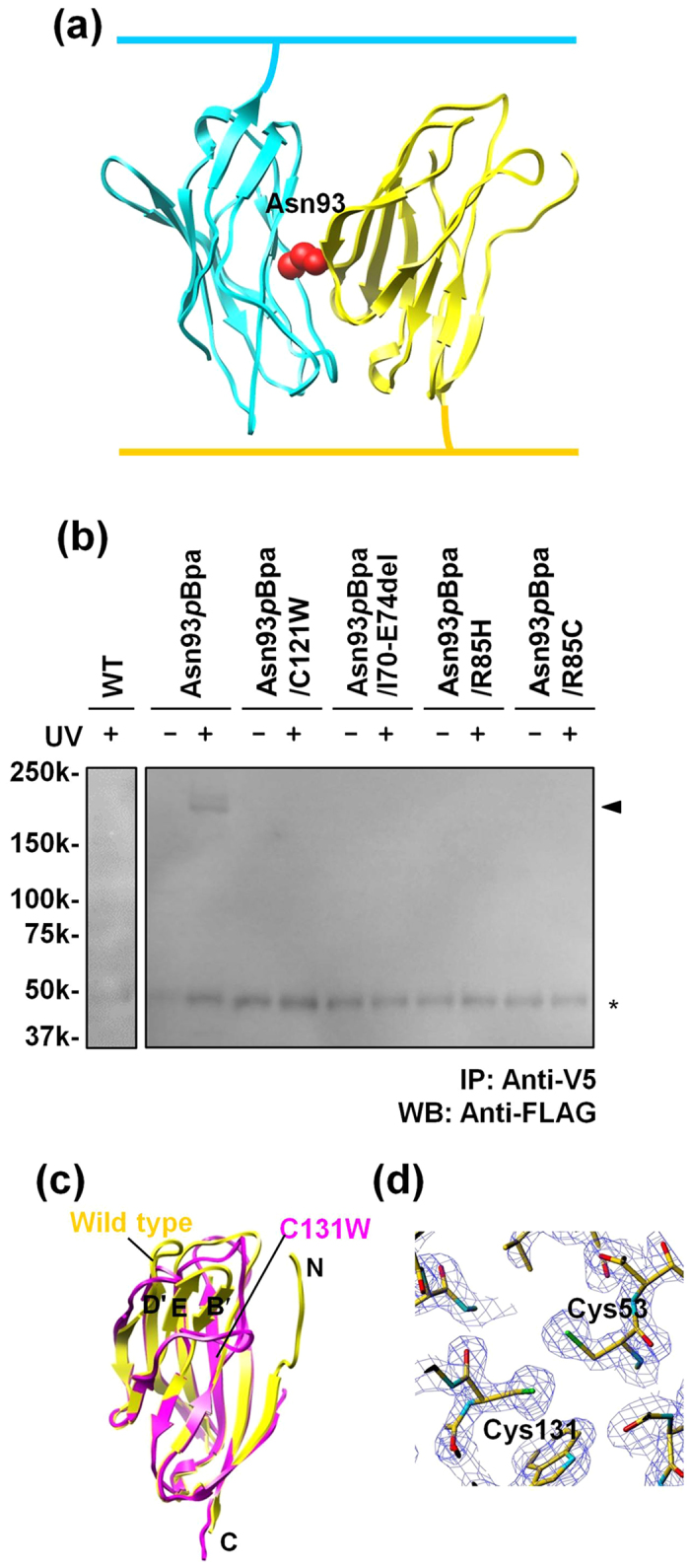
Photo-crosslinking analysis of the *trans* homophilic interaction of β1. (**a**) Mapping of Asn93 substituted by *p*Bpa on the β1 model structure. Asn93 is colored red. (**b**) Photo-crosslinking of the *p*Bpa mutants of β1. The Neuro2A cells separately expressing the *p*Bpa mutants of FLAG- or V5-His-tagged β1 were mixed at a 1:1 ratio, followed by photo-crosslinking. The cell lysates were immunoprecipitated with the anti-V5 antibody, and analyzed by western blotting using the anti-Flag antibody. The triangle indicates the crosslinked β1, and the asterisk indicates the bands corresponding to the anti-V5 antibody, which cross-react with the anti-Flag antibody. (**c**) Comparison of the structures of β4ex (yellow) and the C131W mutant (4MZ3, pink). (**d**) 2Fo−Fc composite omit map (contoured at 1.5 σ) around the intramolecular disulfide bond between Cys53 and Cys131.

In order to clarify that β1 employs a similar *trans* interaction mode to that of β4, we performed the site-directed photo-crosslinking experiment with β1. We mutated the codon for Asn93 (Fig. 5a), corresponding to the crosslinkable residue Glu104 in β4, to the UAG codon (the Asn93*p*Bpa mutant). Two plasmids containing the gene encoding the full-length β1 Asn93*p*Bpa mutant, along with either a Flag or V5-His tag at the C-terminus, were individually introduced into the Neuro2A cells, which were then subjected to photo-crosslinking and immunoprecipitation (Fig. 3e), followed by western blotting. As shown in Supplementary Fig. S5, this mutant was expressed in the presence of *p*Bpa, and the Flag or V5-His tagged *p*Bpa mutants of β1 were detected by western blotting. Furthermore, the crosslinked product was detected by immunoprecipitation with anti-V5 and subsequent western blotting with anti-Flag, in the UV-dependent manner (Fig. 5b). These results indicated that Asn93 is relevant to the *trans* homophilic interface of β1, and the *trans* interaction mode of β1 might be identical to that of β4. The crosslinked product of β1 was detected at approximately 200 kDa, indicating that β1 may also form multimers in the same manner as in the crystal structure of β4.

We also prepared β1 mutants with the Asn93*p*Bpa mutation and one of the GEFS+ mutations, and performed the photo-crosslinking experiment. No crosslinked products were observed for any of the GEFS+ mutations (Fig. 5b), although the mutants were expressed in the presence of *p*Bpa (Supplementary Fig. S5). This observation was consistent with the results of the β1 cell aggregation assay, and further suggested that the GEFS+ mutants could disrupt the *trans* homophilic interaction in cell-cell adhesion. Consequently, we concluded that the *trans* interaction mode is actually employed in β1-mediated cell-cell adhesion.

### Structural mechanisms of the GEFS+ mutations of β1

All of the GEFS+ mutants were expressed well on the cell surface, and affected the *trans* homophilic interaction in cell-cell adhesion, in agreement with previous studies^12^,^13^. Thus, at least in the heterologous system, the impairment of the adhesive function may be a common pathogenic factor among the GEFS+ mutants. Furthermore, our structural information can explain how each of the GEFS+ mutations affects the adhesive function. One of the effects of the C121W mutation may be to disrupt the putative intramolecular disulfide bond to Cys43 in the vicinity of strands B and B′. In the *trans* homophilic interaction, the β-sheet containing strand B directly interacts with the C″–D loop of the other molecule (Fig. 4a,b). The disruption of the intramolecular disulfide bond and the introduction of the bulky Trp residue at this position may sterically disrupt the functional conformation of this β-sheet, thereby altering its interaction with the C″–D loop. This interpretation is supported by the previous structure of the β4 C131W mutant, in which strands B′, E, and D′ are displaced as compared with the present structure^14^ (Fig. 5c). In the present β4 structure, this disulfide bond is not formed at a distance of 3.3 Å (Fig. 5d). This is probably due to the effects of the intense synchrotron radiation or the folding of the recombinant protein under the reducing conditions of the cell-free system. In contrast, the residues deleted in the I70_E74del mutation are located in strand C′, and mainly interact with Leu80–Glu84 in the C″–D loop in the modeled β1 structure (Fig. 4b). Similarly, the R85H and R85C mutations are located in the C″–D loop, and Arg85 forms a salt bridge with Glu82 in the C″–D loop. Therefore, these mutations are both likely to cause abnormal conformations of the C″–D loop, leading to an adverse effect on the *trans* homophilic interaction. The R85H and R85C mutant β1 proteins were reportedly not detectably expressed on the surface of HEK293 cells^22^; however, a more recent analysis revealed the expression of the R85H mutant on the surface of 1610 cells^13^. The GEFS+ mutant proteins may be preferentially expressed on the surfaces of certain cell types, and their effects could vary according to cells, tissues, and/or developmental stages.

## Discussion

Although the crystal structures of the β4, β3 and β2 subunits have recently been reported^14^,^15^,^16^, the structural basis for the functions of the β subunits as CAMs remained unknown. The crystal structures of the β4 molecules lacking the N-terminal segment and the unpaired Cys residue did not provide any clues toward solving the mechanisms of cell-cell adhesion^14^. In the present study, the crystal lattice successfully revealed the antiparallel arrangement of the β4 molecules containing the N-terminal segment and the unpaired Cys residue (Figs 1c and 2a). The antiparallel arrangement is unambiguously related to the interface for the *trans* homophilic interaction of β4 in cell-cell adhesion, based on structure-based mutagenesis and site-directed photo-crosslinking (Figs 2d and 3f). Moreover, the corresponding interface between the β1 molecules (Fig. 4a,b) is involved in the *trans* homophilic interaction for the β1-mediated cell-cell adhesion, and the disruption of this interface is implicated in the GEFS+ pathogenesis (Figs 4d and 5b).

The antiparallel arrangement between the β4 molecules further constitutes the multimeric assembly of β4 in the crystal lattice (Fig. 1d). Moreover, we succeeded in detecting the β4 multimers in cell-cell adhesion by photo-crosslinking, and mutations that disrupt the *trans* homophilic interface between β4 molecules also abolish the multimer formation (Fig. 3f). Consequently, the crystallographic assembly is likely to be relevant to the multimeric assembly of β4 between adhered cells. This multimeric assembly might cooperatively reinforce cell-cell adhesion, even if the individual *trans* interaction between two β4 molecules is not strong enough. Likewise, the participation of β1 multimers in β1-mediated cell-cell adhesion was also detected by photo-crosslinking (Fig. 5b). Moreover, the cell-cell adhesion and the multimer formation of β1 were both disrupted by the GEFS+ mutations (Figs 4d and 5b). The multimeric assembly of β1 is probably formed between adhered cells, which might reinforce cell-cell adhesion. We propose that the impairment of the multimeric assembly of β1, due to the GEFS+ mutations, underlies the abnormal modulation of sodium channels in epilepsy. Thus, the multimeric assembly of β4 and β1 may be a common feature of β subunit-mediated cell-cell adhesion, and may occur with β2 as well.

It was reported that β3 did not mediate the *trans* homophilic interaction^23^. The recently published crystal structure of human β3 revealed that the extracellular domain forms a trimer for a *cis* homophilic interaction^16^. In the β3 structure, the β-sheet surface that corresponds to the interface for the *trans* homophilic interaction of β4 is located inside the trimeric structure, and therefore cannot interact with the C″–D loop of another β3 molecule. Thus, the lack of the *trans* homophilic interaction is probably due to the trimerization of β3.

β4 contains three conserved Cys residues, among which the unpaired Cys residue (Cys58) forms a disulfide bond with the α subunit in *cis*. The Cys58 residue is located near the N-terminal segment, and is far away from the *trans* adhesive interface in the arrangement of the β4 molecules (Fig. 1d). Therefore, Cys58 of the adhered β4 molecule is accessible to the α subunit, indicating that the α–β4 complex may contribute to cell-cell adhesion.

Indeed, Asn113 is one of the potential glycosylation sites. However, when the full-length β4 molecule was expressed in CHO cells, the molecular weight of the L50S/R94D/V95S/N113D mutant was approximately the same as that of the WT both before and after deglycosylation, as determined by a western blot analysis (Supplementary Fig. S6a). Therefore, the Asn113 residue is presumably not glycosylated in CHO cells, and the modification of this residue is not relevant to the cell aggregation properties of the L50S/R94D/V95S/N113D and N113D mutants. In addition, Ser48 lies close to Asn45, which is another potential glycosylation site. As shown in Supplementary Fig. S6b, Ser48 is located in the trans homophilic interface, whereas the side chain of Asn45 is oriented oppositely to the interface. Thus, the putative glycosylation of Asn45 seems to have only a negligible effect on cell adhesion due to β4.

The cell aggregation assay has been the most commonly used method to evaluate the adhesive properties of cell adhesion molecules. However, this assay is not sufficient to directly detect the *trans* homophilic and heterophilic interactions between cells. In contrast, the site-directed photo-crosslinking technique, using the photo-reactive amino acid, is useful for analyses of protein-protein interactions at the interface of interest in a variety of host systems, and is applicable to membrane proteins. In this study, the *trans* homophilic interactions of membrane proteins between adhered cells were successfully detected by the photo-crosslinking experiments in mammalian cells, and this is the first description of these phenomena, to the best of our knowledge. Our method is generally applicable to other membrane proteins, to capture the protein-protein interactions mediating cell-cell communications.

## Methods

### Protein preparation by the cell-free system and crystallization

The DNA fragment encoding residues 30–160 of the mouse β4 (GenBank accession number: BK001031) subunit was cloned into the expression vector pCR2.1 TOPO (Life Technologies), as fusions with an N-terminal histidine tag and a TEV protease cleavage site (amino acid numbering starts from the initiator methionine residue). The proteins were produced by the *Escherichia coli* cell-free method^24^. The reaction solutions were centrifuged at 16,000 *g* and 4 °C for 20 min, and the supernatants were loaded onto a HisTrap column (all chromatography materials were purchased from GE Healthcare Bio-Sciences), equilibrated with 20 mM Tris-HCl buffer (pH 8.0), containing 1.0 M NaCl, 10% glycerol, and 20 mM imidazole. After washing the column with the buffer, the His-tagged proteins were eluted with 20 mM Tris-HCl buffer (pH 8.0), containing 500 mM NaCl, 10% glycerol, and 500 mM imidazole. The sample buffer was exchanged to 20 mM Tris-HCl buffer (pH 8.0), containing 1.0 M NaCl, 10% glycerol, and 20 mM imidazole, with a HiPrep 26/10 desalting column, and the His-tag was cleaved by TEV protease at 4 °C overnight. To remove the uncleaved protein, the reaction solutions were loaded onto the HisTrap column, as described above. The flow-through fractions were collected, and desalted on a HiPrep 26/10 desalting column, equilibrated with 20 mM Tris-HCl buffer (pH 8.5), containing 20 mM NaCl and 10% glycerol. The pooled fractions were loaded onto a Mono Q column, equilibrated with 20 mM Tris-HCl buffer (pH 8.0), containing 10 mM NaCl and 10% glycerol. The proteins were eluted with a linear gradient of 10 mM to 1.0 M NaCl. Finally, the purified protein fractions were loaded on a HiLoad 16/60 Superdex 75 column, equilibrated with 20 mM Tris-HCl buffer (pH 8.0), containing 150 mM NaCl and 10% glycerol. The purified proteins were concentrated to 8–11 mg/ml. The crystals were obtained in a drop composed of 0.5 μl protein solution and 0.5 μl reservoir solution, containing 30% (*w*/*v*) polyethylene glycol (PEG) 1500, equilibrated against 80 μl of the reservoir solution by the sitting drop vapor diffusion method at 20 °C.

### Determination of the structures of β4

Before data collection, the crystal was transferred to mother liquor containing 15% glycerol as a cryoprotectant, and flash-cooled in a nitrogen-gas stream at 100 K. Diffraction data were collected on BL26B2 via the Mail-in data collection system^25^ at SPring-8 Center, at a temperature of 100 K. The data set was integrated and scaled using the HKL2000 and SCALEPACK software packages^26^. The structure was solved by molecular replacement using the CCP4 package^27^, by employing the previously determined myelin P0 structure (Protein Data Bank accession no. 1NEU^28^) as a search model. The structure was modeled by the program ARP/wARP^29^, and refined by rigid-body fitting followed by the simulated-annealing protocol implemented in the program CNS^30^. Model building and further refinement were performed with the programs COOT^31^ and PHENIX^32^. Data collection and refinement statistics are shown in Supplementary Table S1. The atomic coordinates and experimental data have been deposited in the Protein Data Bank, under the accession code 5AYQ.

### Antibodies

Primary antibodies were as follows: rabbit polyclonal anti-β1 and anti-β4^33^; rabbit polyclonal anti-β1ex (Abcam ab107370); mouse monoclonal anti-Na^+^/K^+^ ATPase (Abcam, ab7671); mouse monoclonal anti-V5-HRP (Life Technologies, R961-25); polyclonal anti-V5 (Funakoshi, GeneTex, GTX117997); and anti-Flag M2-HRP (SIGMA, A8592). The secondary antibodies included chicken anti-rabbit IgG (H+L)-Alexa Fluor 647 (Life Technologies, A21443), which was used for immunofluorescence, and HRP-conjugated anti-mouse and anti-rabbit IgG (GE Healthcare Bio-Sciences, NA931 and NA934, respectively), which were used for western blotting.

### Western blotting

Western blotting was performed as described previously^33^,^34^. The cells were washed twice with PBS, and lysed in 25 mM Tris-HCl buffer (pH 8.0), containing 150 mM NaCl, 0.1% SDS, 1.0% Triton X-100, 0.5% deoxycholic acid, and protease inhibitors. The supernatants, collected after centrifugation at 500 *g* for 10 min at 4 °C, were heated at 95 °C for 3 min in SDS sample buffer containing 5 mM DTT, fractionated on 5–20% polyacrylamide gels, and probed with the indicated specific monoclonal antibodies. Full-length blots are presented in Supplementary Fig. S7.

### Immunofluorescence microscopy

The cells were grown on glass bottom plates coated with collagen, and induced with doxycycline as described above. The cells were then fixed with PBS, containing 4% paraformaldehyde and 0.12 M sucrose, for 20 min. The cells were incubated with PBS containing 0.1% gelatin for 5 min, and then incubated with the anti-β1ex antibody (Abcam) for 1 h at room temperature. After the primary antibody incubation, the cells were subsequently incubated with an Alexa Fluor 647-conjugated secondary antibody for 30 min at room temperature. Cell nuclei were labeled with Hoechst 33342. The cells were mounted with a SlowFade antifade kit (Molecular Probes), and viewed using a Zeiss LSM 510 confocal microscope.

### Cell aggregation assay

The cell aggregation assay was performed according to the method of Agarwala *et al*.^35^. The stable cell lines were grown to 70–80% confluency, and induced with doxycycline as described above. After washing twice with PBS, the cells were incubated with PBS containing 2 mM EDTA (pH 7.5) for 15 min at 37 °C, and then dispersed by gentle pipetting. The cells were suspended in DMEM at 1.0 × 10^6^ cells/ml for CHO cells, and 0.3 × 10^6^ cells/ml for Neuro2a cells, and then transferred to polystyrene tubes. For the assay using CHO stable cell lines, the cell suspensions were incubated at 37 °C, and aliquots were counted at 30 min intervals with a hemocytometer, after mixing by several gentle inversions. For the assay using the Neuro2a stable cell lines, the cell suspensions were incubated at 37 °C for 30 min, and then counted, as cell damage gradually occurred over 60 min of incubation.

### Site-specific crosslinking

The full-length mouse β4 and β1 genes, containing either a C-terminal FLAG tag or V5-His tag, were mutated by changing the codons encoding Glu104 in β4 and Asn93 in β1 to TAG codons. The CHO cells expressing β4 and the Neuro2A cells expressing β1 were plated in 100 mm cell culture dishes. The cells were transfected with 6 μg of the β4 or β1 plasmid plus 6 μg of the pcpBpaRS ver.1 plasmid, encoding the transfer RNA (tRNA) specific to the UAG stop codon and an engineered aminoacyl-tRNA synthetase specific to *p*-benzoyl-L-phenylalanine (*p*Bpa, Watanabe Chemical Industries)^18^,^19^, using Lipofectamine LTX. After an incubation for 16 hours in medium supplemented with 0.5 mM *p*Bpa, the cells were detached from the culture dishes with PBS containing 2 mM EDTA (pH 7.5), and collected by centrifugation at 1,000 *g* for 10 min at 20 °C. The cells were then suspended in DMEM, and mixed at a 1:1 ratio in a cell culture dish. After an incubation for 3 hours at 37 °C in a CO_2_ incubator, the cells were exposed to UV light (375 nm) for 30 min on ice, and lysed in an appropriate volume of 60 mM Tris-HCl buffer (pH 7.5), containing 180 mM NaCl, 1.25% Triton X-100, 6 mM EDTA (pH 7.5), and protease inhibitors. The supernatants were collected after centrifugation at 14,000 *g* for 30 min at 4 °C, and incubated with protein A- or protein G-magnetic beads (Thermo Fisher Scientific Inc. and GE Healthcare Bio-Sciences), immobilized with the polyclonal anti-V5 antibody, by rotating end-over-end overnight at 4 °C. The beads were then washed extensively with 50 mM Tris-HCl buffer (pH 7.5), containing 150 mM NaCl, 0.1% Triton X-100, 5 mM EDTA (pH 7.5), and protease inhibitors. The proteins were eluted by the addition of 50 μl of SDS sample buffer containing 5 mM DTT, incubated at 95 °C for 3 min, and analyzed by western blotting using the indicated antibodies.

## Additional Information

**How to cite this article**: Shimizu, H. *et al*. Structure-based site-directed photo-crosslinking analyses of multimeric cell-adhesive interactions of voltage-gated sodium channel β subunits. *Sci. Rep.* **6**, 26618; doi: 10.1038/srep26618 (2016).

## Supplementary Material

Supplementary Information
