# Supplementary material for: Structure-based site-directed photo-crosslinking analyses of multimeric cell-adhesive interactions of voltage-gated sodium channel β subunits
Source: Sci Rep. 2016 May 24;6:26618. doi: 10.1038/srep26618 (PMC4877568; doi:10.1038/srep26618)
Supplement: Supplementary Information [file srep26618-s1.pdf]

Supplementary Information

Structure-based site-directed photo-crosslinking analyses of multimeric cell-adhesive interactions of voltage-gated sodium channel β subunits

Hideaki Shimizu[1,2,3], Haruko Miyazaki[3,4,5], Noboru Ohsawa[1,2], Shisako Shoji[1,2], Yoshiko Ishizuka-Katsura[1,2], Asako Tosaki[3], Fumitaka Oyama[3,6], Takaho Terada[1,7], Kensaku Sakamoto[1,2], Mikako Shirouzu[1,2], Shun-ichi Sekine[1,2] , Nobuyuki Nukina[3,4,5] and Shigeyuki Yokoyama[1,7]

[1] RIKEN Systems and Structural Biology Center, Tsurumi, Yokohama 230-0045, Japan

[2] RIKEN Center for Life Science Technologies, Tsurumi, Yokohama 230-0045, Japan

[3] Laboratory for Structural Neuropathology, RIKEN Brain Science Institute, Wako, Saitama 351-0198, Japan

[4] Department of Neuroscience for Neurodegenerative Disorders, Juntendo University Graduate School of Medicine, Tokyo 113-8421, Japan

[5] Laboratory of Structural Neuropathology, Doshisha University Graduate School of Brain Science, 1-3 Tatara Miyakodani, Kyotanabe-shi, Kyoto 610-0394 Japan

Tel&Fax:81-774-65-7211 [6] Department of Chemistry and Life Science, Kogakuin University, Hachioji, Tokyo 192-0015, Japan.

[7] RIKEN Structural Biology Laboratory, Tsurumi, Yokohama 230-0045, Japan

Supplementary Table S1

Data collection and refinement statistics.

**Data collection**	
Space group	*C*2
Unit-cell parameters (Å, °)	103.7, 59.3, 42.4, 90, 106, 90
Resolution (Å)	100–1.7 (1.76–1.7)
R_{sym} (%)	9.2 (89.6)
I /σ (I)	33.4 (2.5)
Completeness (%)	99.9 (100)
Redundancy	7.4 (7.4)
$CC_{1/2}$	0.999 (0.630)
$R_{p.i.n.}$	0.039 (0.514)
**Refinement**	
Resolution (Å)	29.8–1.7 (1.73–1.7)
No. reflections	27530 (1608)
R_{work}/ R_{free} (%)	20.3/23.1 (27.4/29.1)
No. atoms	
Protein	1883
Water	230
B-factor (Å^2)	
Protein	26.1
Water	35.2
R.m.s. deviations	
Bond lengths (Å)	0.005
Bond angles (°)	0.855
Ramachandran plot	
Favored (%)	97.0

Outliers (%)	0

One crystal was used for each dataset. Values in parentheses are for the highest-resolution shell.

Supplementary Methods

Cell culture and transfection

For stable expression, the Flp-In CHO cell lines expressing mouse β4 and the Neuro2a/FRT/TR cell lines expressing mouse β1 were generated, according to the manufacturer's protocol (Life Technologies). The Flp-In CHO cell lines were cultured in Ham's F12 medium, and the Neuro2a/FRT/TR cell lines were cultured in DMEM. The DNAs were cloned into the expression vector pcDNA5/FRT, and transfected into cells using LipofectAMINE 2000 (Life Technologies). The transfected cells were selected with hygromycin (400–500 μg/ml for CHO cells and 100-200 μg/ml for Neuro2a cells). The protein expression was induced by adding 1.0 μg/mL doxycycline to the medium, for 16 h for CHO cells and 24 h for Neuro2a cells, and the clones expressing similar levels of β subunits were selected by limited dilution, western blotting and immunocytochemical analyses. For transient expression, the CHO cells were cultured in Ham's F12 medium, and the Neuro2A cells were cultured in DMEM. The DNAs were cloned into the expression vector pcDNA3.1/V5-His-TOPO, and transfected into cells using LipofectAMINE LTX. All cells were originally sourced from ATCC, but not authenticated. Plasmocin treatment has been employed routinely to these cell lines.

Deglycosylation assay

The CHO cells stably expressing β4s were induced with doxycycline, as described in the METHODS section. The cells were washed twice with PBS, detached from the culture dishes using PBS containing 2 mM EDTA (pH 7.5), and lysed in 50 mM Tris-HCl buffer (pH 7.5), containing 150 mM NaCl, 1.0% Triton-X, and protease inhibitors. Supernatants, collected after centrifugation at 14,000 g for 10 min at 4 °C, were incubated with peptide-N-glycosidase F (PNGase F) at 4 °C overnight, and then analyzed by western blotting.

Cell surface biotinylation

The cells were biotinylated and isolated with a Cell Surface Protein Isolation kit (Pierce 89881), according to the manufacturer's protocol. Samples were analyzed by western blotting.

Statistical analysis

All data are presented as the means ± S.E.M., and the one-way ANOVA following the *post hoc* tests (Tukey's and Bonferroni's) was performed, as described in the figure legends. All data have normal distributions (tested by the Shapiro-Wilk test and the D'Agostino-Pearson omnibus tests) and equal variances (tested by the F-test and Bartlett's test).

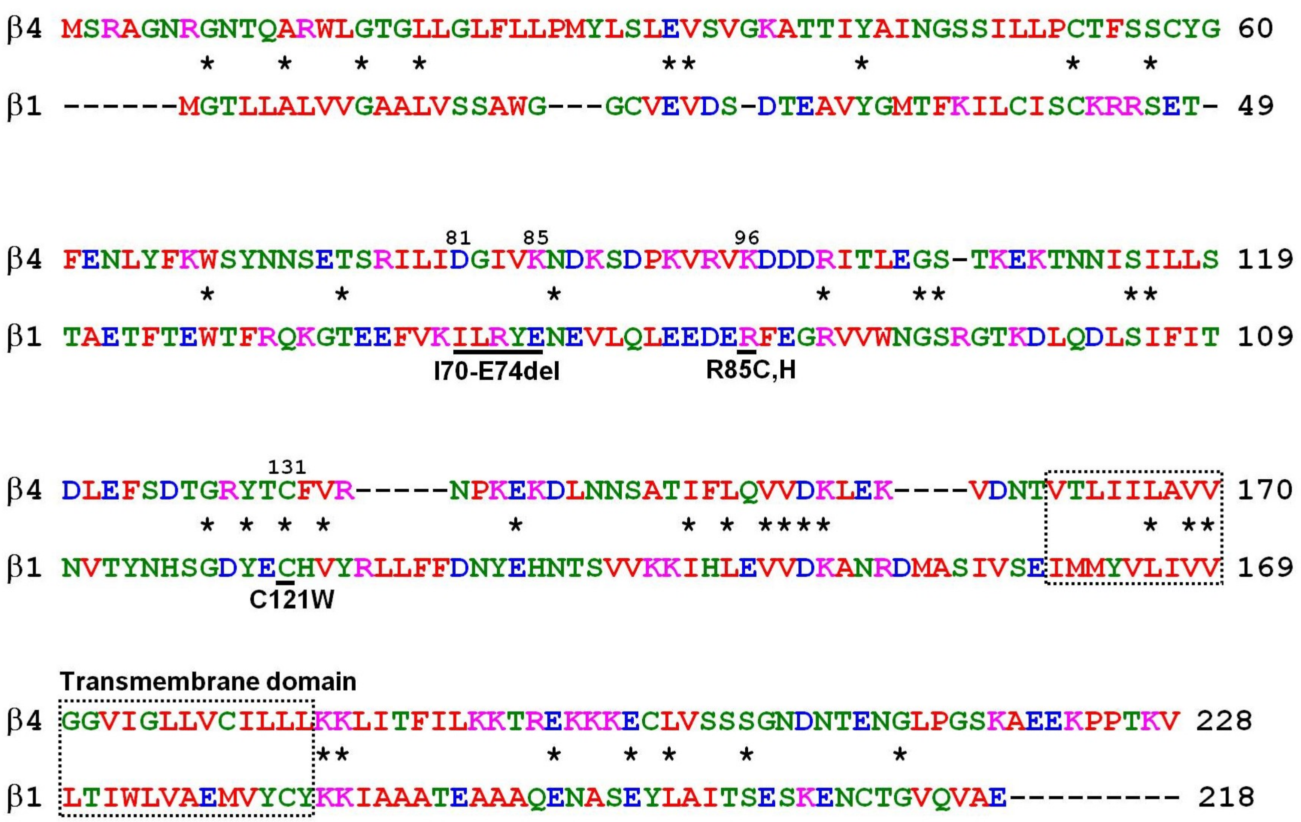

Supplementary Figure S1 Amino acid sequences of the mouse β4 and β1 subunits.

Sequence alignment of the mouse β4 and β1 subunits, accomplished using ClustalW (EMBL-EBI). Conserved types of amino acids are color-coded according to the ClustalW program (AVFPMILW are colored red, DE are blue, RK are magenta, and STYHCNGQ are green).

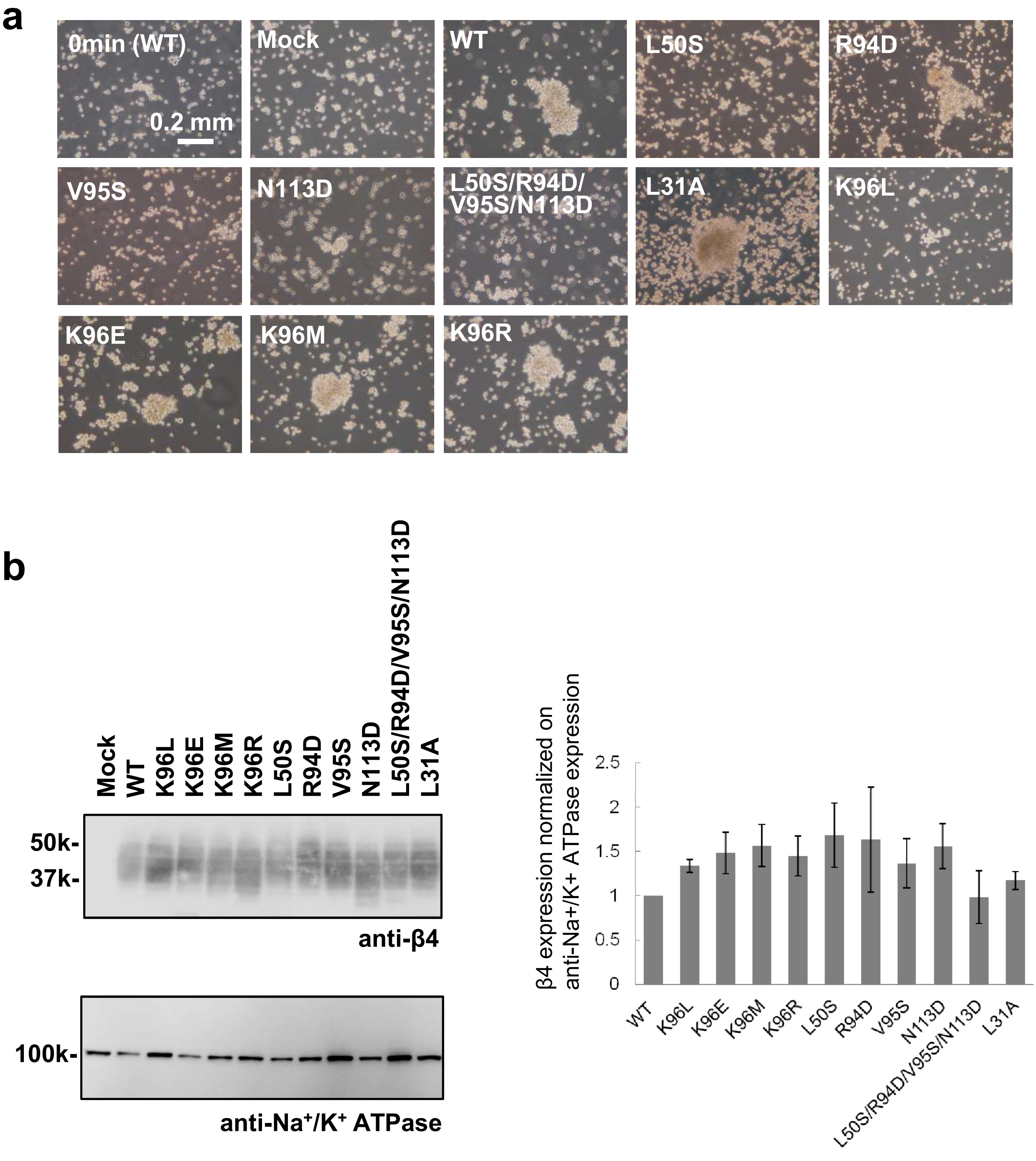

Supplementary Figure S2 Cell aggregation and cell surface biotinylation assays of the β4 mutants.

a Cell aggregation patterns of CHO cells stably expressing the WT and mutants of β4 after a 120 min incubation.

b Cell surface biotinylation of CHO cells stably expressing the WT and mutants of β4, analyzed by western blotting. Representative western blots using anti-β4 (upper left), anti-Na+/K+ ATPase (lower left), and quantification of β4 bands normalized on the corresponding anti-Na+/K+ ATPase bands (right). Data are means ± S.E.M., n = 3.

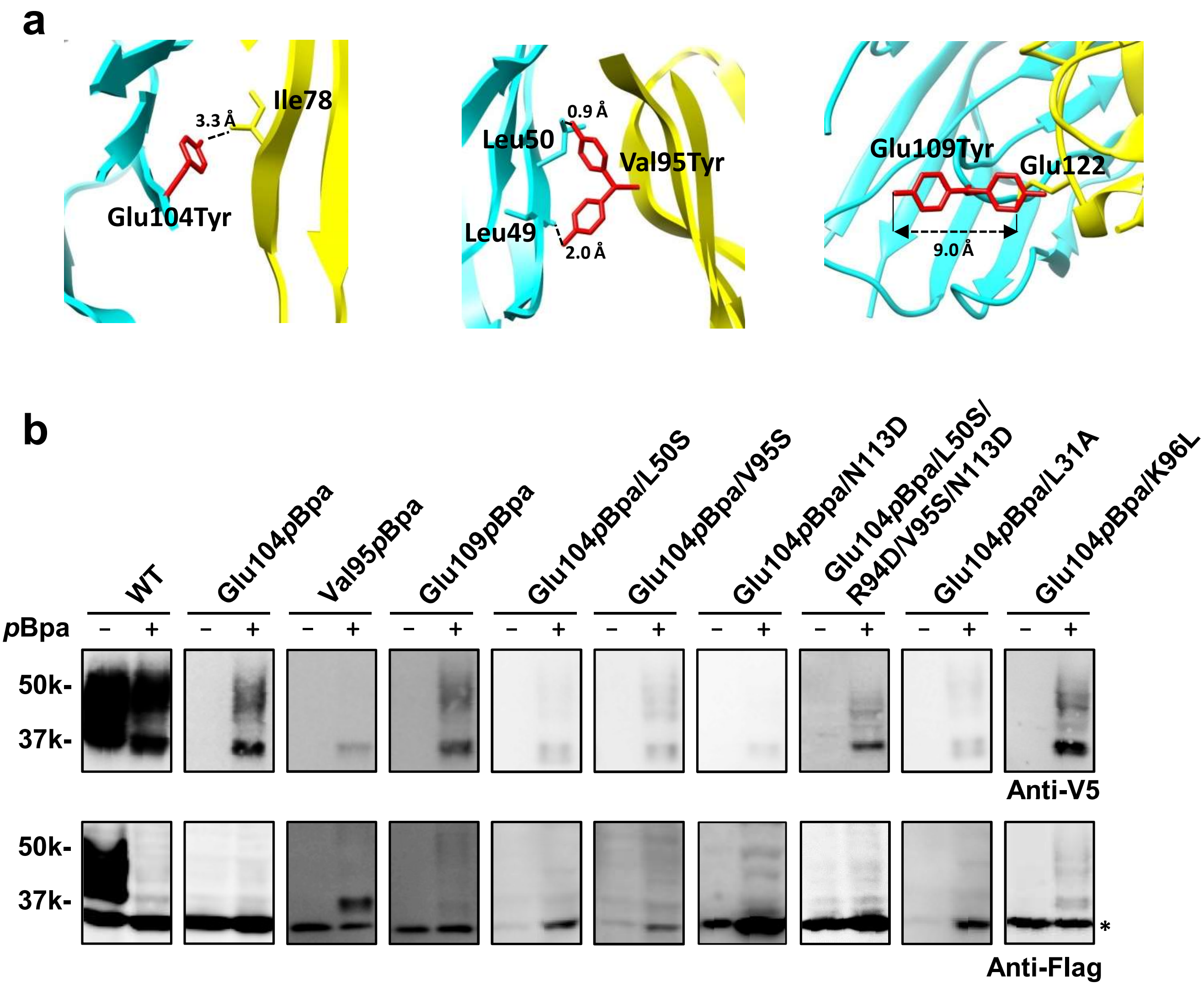

Supplementary Figure S3 Photo-crosslinking analysis of β4.

a The imaginary structures of the Glu104*p*Bpa, Val95*p*Bpa, and Glu109*p*Bpa mutants. Each *p*Bpa was replaced by a tyrosine residue, and sterically reasonable rotameric conformations are displayed.

b Incorporation of *p*Bpa into β4. CHO cells transfected with the WT and *p*Bpa mutants of V5-His- (upper) and Flag-tagged (lower) β4 were cultured in the presence or absence of *p*Bpa for 24 h. Cell lysates were analyzed by western blotting, using the indicated antibodies. The asterisk indicates nonspecific bands that react with the anti-Flag antibody.

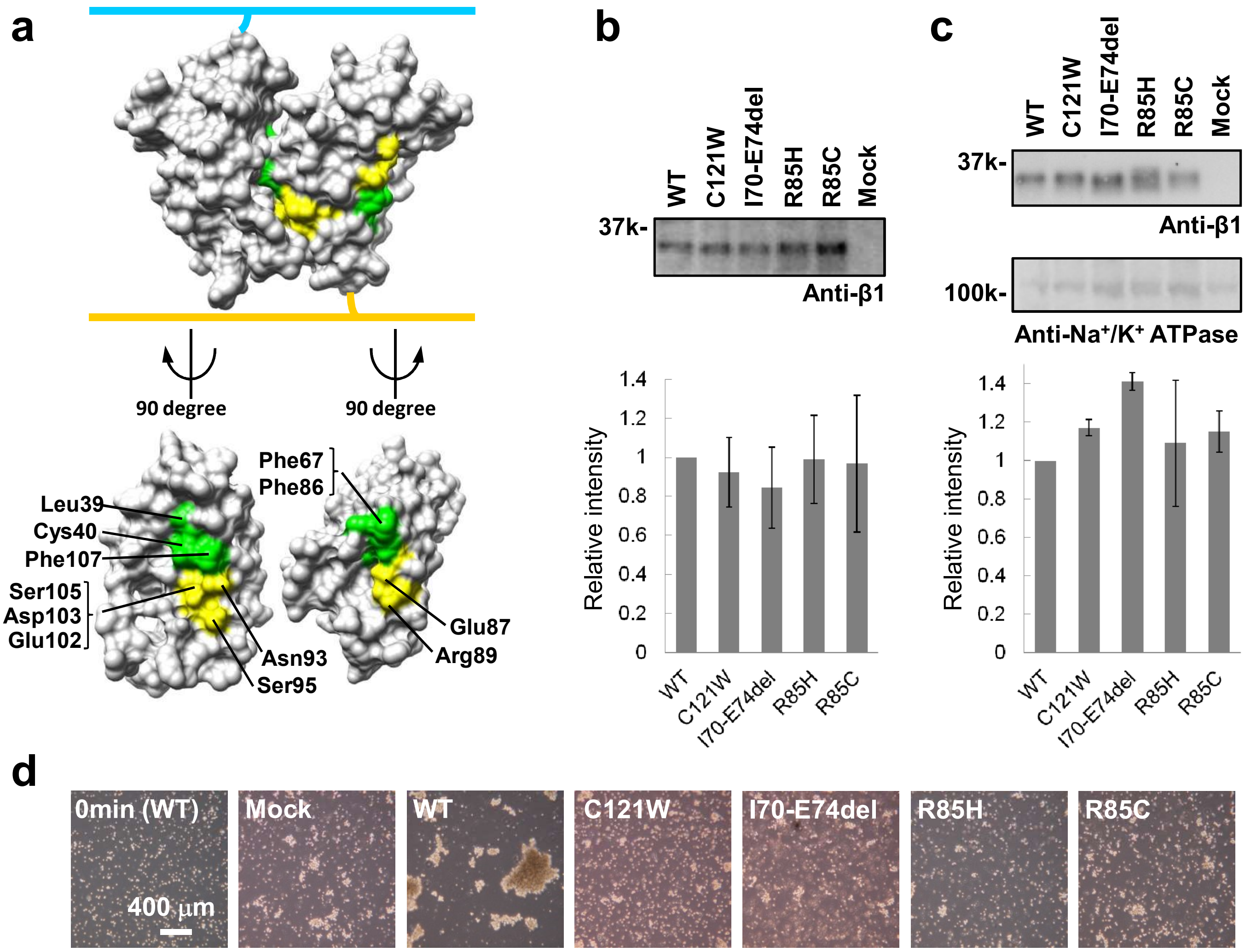

Supplementary Figure S4 Analysis of the *trans* homophilic interaction of β1.

a Surface representations of the contact areas within the *trans* homophilic interface of the β1 model structure. The surface-exposed hydrophobic residues are colored green, and hydrophilic residues are yellow. Side view in the same orientation as in Fig. 4a (upper), and view of the contact surface of the *trans* homophilic interface (lower).

b Western blot analysis of Neuro2A cells stably expressing the WT and GEFS+ mutants of β1. Representative western blot (upper), and quantification of bands (lower). Data are means ± S.E.M., n = 3.

c Cell surface biotinylation of Neuro2A cells stably expressing the WT and GEFS+ mutants of β1, analyzed by western blotting. Representative western blots using anti-β1 (upper), anti-Na+/K+ ATPase (middle), and quantification of bands using anti-β1 (lower). Data are means ± S.E.M., n = 3.

d Cell aggregation patterns after a 30 min incubation.

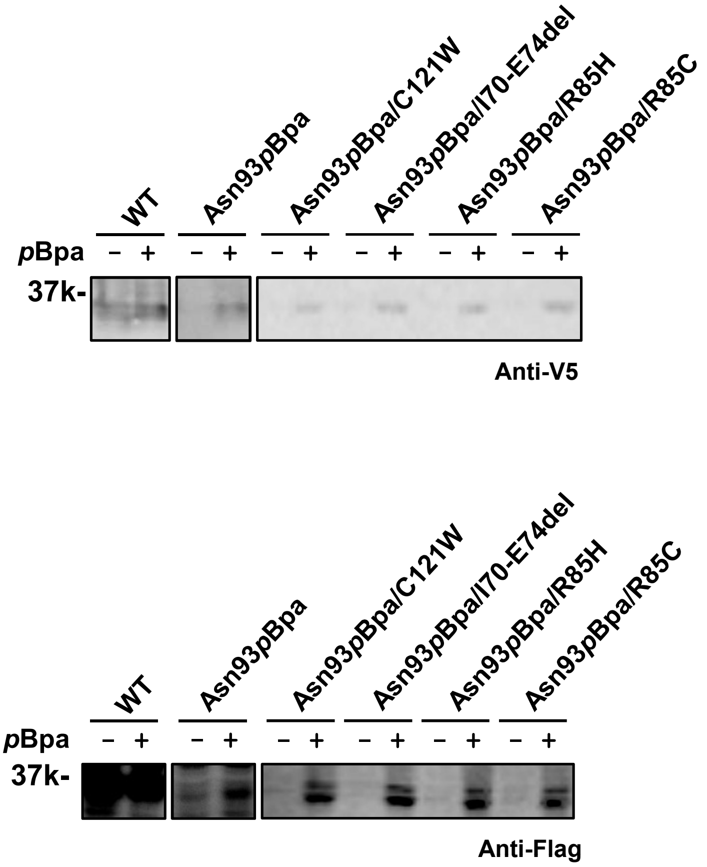

Supplementary Figure S5 Incorporation of *p*Bpa into β1.

Neuro2A cells transfected with the WT and *p*Bpa mutants of V5-His- (upper) and Flag-tagged (lower) β1 were cultured in the presence or absence of *p*Bpa for 24 h. Cell lysates were analyzed by western blotting, using the indicated antibodies.

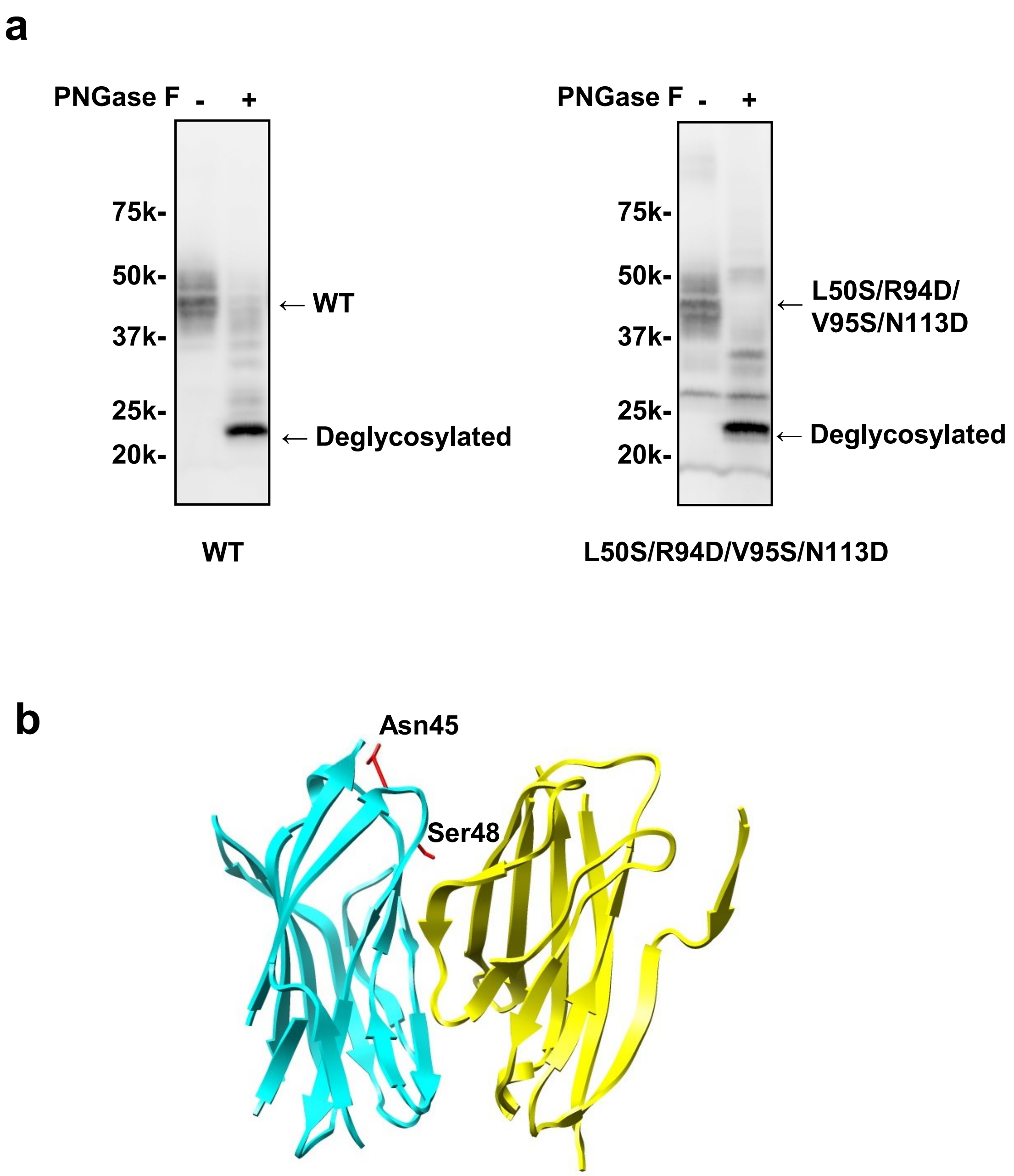

Supplementary Figure S6 Putative glycosylation sites in the extracellular domains of β4.

a Deglycosylation of the WT and the L50S/R94D/V95S/N113D mutant of β4, stably expressed in CHO cells. Cell lysates were treated with or without peptide-*N*-glycosidase F (PNGase F). The samples were then analyzed by western blotting, using the anti-β4 antibody.

b Location of the Asn45 and Ser48 residues.

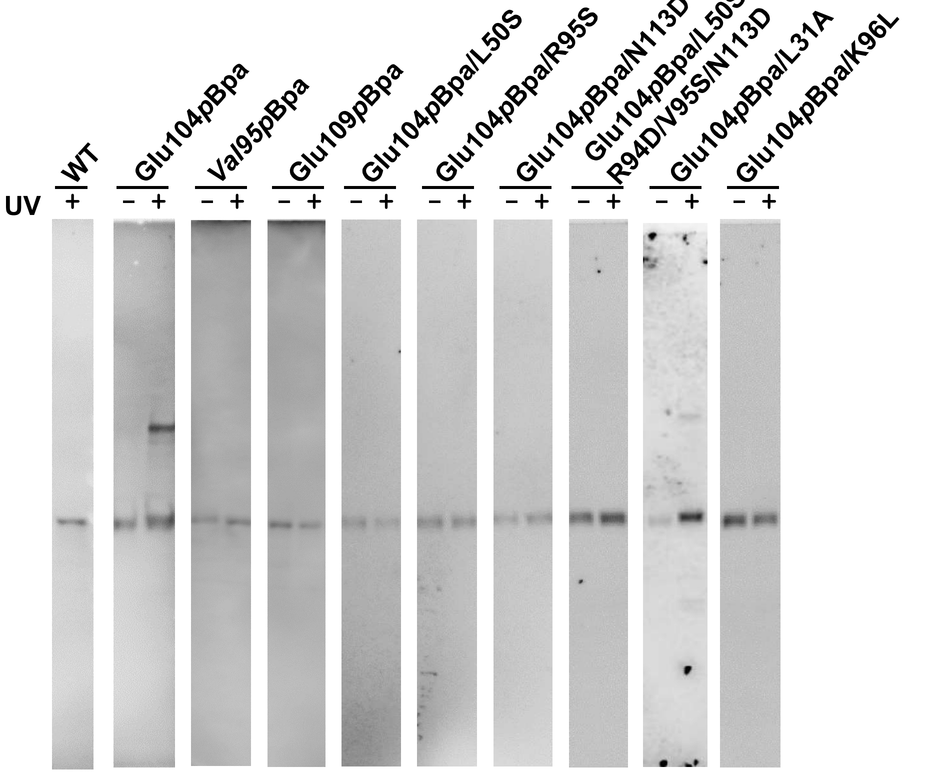

Fig. 3f

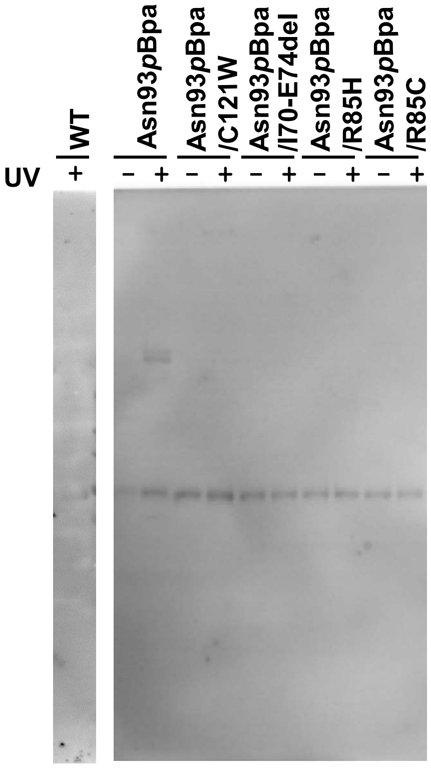

Fig. 5b

Supplementary Figure S7 Full-length blots and gels presented in the main figures.
